# Subcutaneous Immunoglobulin 16.5% (Cutaquig®) in Primary Immunodeficiency Disease: Safety, Tolerability, Efficacy, and Patient Experience with Enhanced Infusion Regimens

**DOI:** 10.1007/s10875-023-01509-4

**Published:** 2023-05-09

**Authors:** Sudhir Gupta, James DeAngelo, Isaac Melamed, Jolan E. Walter, Ai-Lan Kobayashi, Tracy Bridges, J. Wesley Sublett, Jonathan A. Bernstein, Alan Koterba, Michael Manning, Joanna Maltese, Sonja Hoeller, Eva Turpel-Kantor, Huub Kreuwel, Roger H. Kobayashi

**Affiliations:** 1 University of California, Irvine, CA USA; 2 University of Pittsburgh, Pittsburgh, PA USA; 3 IMMUNOe Research Center, Centennial, CO USA; 4 University of South Florida, St. Petersburg, FL USA; 5 Midland Pediatrics, Papillion, NE USA; 6 Allergy and Asthma Clinics of Georgia, Albany, GA USA; 7 Family Allergy & Asthma, Louisville, KY USA; 8 University of Cincinnati, Cincinnati, OH USA; 9 Allergy Associates, North Palm Beach, FL USA; 10 Medical Research of Arizona, Scottsdale, AZ USA; 11 Octapharma USA, Inc, Paramus, NJ USA; 12 Octapharma AG, Vienna, Austria; 13 University of California, Los Angeles, CA USA

**Keywords:** Subcutaneous immunoglobulin (SCIG), Primary immunodeficiency disease, Cutaquig, High infusion volume, High infusion rate, Every other week dosing, Biweekly dosing

## Abstract

**Purpose:**

To achieve reductions in infusion time, infusion sites, and frequency, a prospective, open-label, multicenter, Phase 3 study evaluated the safety, efficacy, and tolerability of subcutaneous immunoglobulin (SCIG) 16.5% (Cutaquig®, Octapharma) at enhanced infusion regimens.

**Methods:**

Three separate cohorts received SCIG 16.5% evaluating volume, rate, and frequency: Cohort 1) volume assessment/site: up to a maximum 100 mL/site; Cohort 2) infusion flow rate/site: up to a maximum of 100 mL/hr/site or the maximum flow rate achievable by the tubing; Cohort 3) infusion frequency: every other week at twice the patient’s weekly dose.

**Results:**

For Cohort 1 (n = 15), the maximum realized volume per site was 108 mL/site, exceeding the currently labeled (US) maximum (up to 40 mL/site for adults). In Cohort 2 (n = 15), the maximum realized infusion flow rate was 67.5 mL/hr/site which is also higher than the labeled (US) maximum (up to 52 mL/hr/site). In Cohort 3 (n = 34), the mean total trough levels for every other week dosing demonstrated equivalency to weekly dosing (p value = 0.0017). All regimens were well tolerated. There were no serious bacterial infections (SBIs). Most patients had mild (23.4%) or moderate (56.3%) adverse events. The majority of patients found the new infusion regimens to be better or somewhat better than their previous regimens and reported that switching to SCIG 16.5% was easy.

**Conclusions:**

SCIG 16.5% (Cutaquig®), infusions are efficacious, safe, and well tolerated with reduced infusion time, fewer infusion sites, and reduced frequency. Further, the majority of patients found the new infusion regimens to be better or somewhat better than their previous regimens.

## Introduction

In pediatric and adult patients with primary immunodeficiency disease (PIDD) who have major deficiencies in antibody synthesis, immunoglobulin G [IgG] is used therapeutically for replacement therapy (IgRT) [1–3].The principal IgG infusion methods used include intravenous administration (IVIG) and subcutaneous administration (SCIG). Over the years, it has been found that SCIG offers advantages over IVIG from both the patient and physician perspective including stable serum IgG levels [4–6], fewer systemic side effects [5, 7–9], improved compliance [5, 10–12], and reduced hospitalizations [10, 12].

As patients and infusion providers have become more proficient and familiar with SCIG, procedures to shorten infusion time and frequency, and reduce the efforts associated with infusion tasks, have been increasingly explored. Obvious means of improving infusion time, convenience, and effort would include faster subcutaneous infusion rates, infusion of larger volumes, less frequent infusions, and more convenient and efficient means of self infusion.

A number of studies have looked at decreasing infusion times [13–15], larger volumes [16, 17], and decreased frequency [18–21]. The aim of this prospective, open-label, multicenter, Phase 3 study [SCGAM-06; NCT03939533] was to evaluate all three parameters in one comprehensive trial to determine whether it is reasonable to pursue more convenient IgRT by the subcutaneous method. To this end, the efficacy and safety of SCIG 16.5% (Cutaquig®, Octapharma AG), at enhanced infusion regimens, was evaluated. SCIG 16.5% is currently approved for the treatment of PIDD in the US and Europe and for secondary immunodeficiencies in Europe and Canada [22–24]. At present, the maximum infusion volume for SCIG 16.5% in the US is 40 mL/site (for adults ≥ 17 years), 29 mL/site (for ages 7–16 years), and 15.5 mL/site (for ages 2–6 years). In the EU no maximum infusion volumes are specified; however, doses over 30 mL may be divided according to patient preference in adults [23]. The current maximum infusion rate for SCIG 16.5% in the US is 52 mL per hour per site for adults (≥ 17 years) and 25 mL per hour per site for children (2–16 years) [22]. In the EU, the recommended initial administration rate is 15 ml/h/site. For subsequent infusions, if well tolerated, it can be increased up to 25 ml/h/site [23].

Previous clinical studies have provided guidance on the infusion parameters of SCIG 16.5% when switching adult and pediatric PIDD patients from IVIG to SCIG [7, 8]. These studies, as well as other evaluations of similar SCIG products [16, 17], have suggested that higher infusion parameters may potentially be well-tolerated. The current study was designed to evaluate enhanced infusion regimens that may lead to increased patient compliance and treatment satisfaction by defining greater infusion flexibility. By increasing the infusion volume per site, the benefit is a reduction in the required number of infusion sites. The advantage of increasing the infusion flow rate is reduced overall time for infusion. In addition, reduction in the total number of infusions, by infusing on an every other week basis, reduces the overall number of infusions required.

## Methods

### Study Design

The results reported herein are from the final analysis of the SCGAM-06 study [NCT03939533] that concluded in 2022. The study was designed to evaluate enhanced infusion regimens that would allow for greater administration flexibility, potentially leading to increased patient compliance and treatment satisfaction. The co-primary objectives of this study were: 1) comparison of total IgG trough levels from weekly infusions to every other week infusions; 2) determination of the safety and tolerability of SCIG 16.5% when administered at increased volume at each infusion site; 3) determination of the safety and tolerability of SCIG 16.5% when administered at increased flow rate at each infusion site; and 4) determination of the safety and tolerability of SCIG 16.5% when administered every other week. Additional objectives included evaluation of quality of life (QoL) measures and patient satisfaction.

**Fig. 1:**
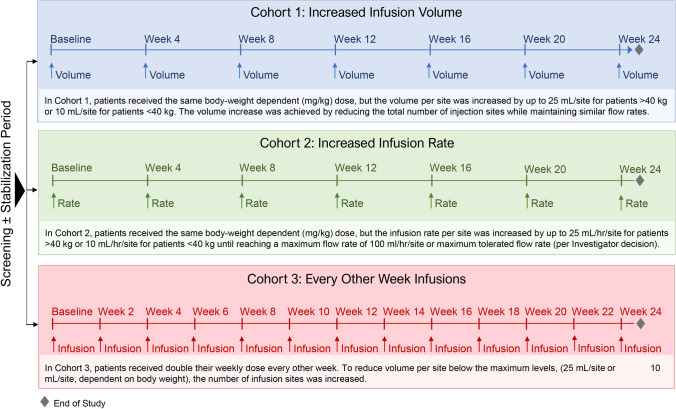
SCGAM-06 study design

Three separate cohorts received SCIG 16.5% with alternative infusion regimens evaluating volume, rate, and frequency: 1) volume assessment/site: up to a maximum 100 mL/site; 2) infusion flow rate/site: up to a maximum of 100 mL/hr/site, or the maximum flow rate achievable by the tubing; 3) infusion frequency: every other week at the equivalent of twice the patient’s body-weight dependent [mg/kg] weekly dose (Fig. 1).

Safety was evaluated based on the occurrence of treatment-emergent adverse events (TEAEs), including infusion-site reactions (ISRs). An Independent Data Monitoring Committee (IDMC) periodically reviewed relevant data throughout the study with an emphasis on AEs including serious adverse events (SAEs) and thromboembolic events. Efficacy evaluations were based on the maintenance of IgG trough levels, prevention of infections (including serious bacterial infections [SBIs]), antibiotic use (number of days and annual rate), and QoL as measured by the 36-Item Short-Form Questionnaire (SF-36) in patients ≥ 14 years of age and the SF-10 in patients ≤ 13 years of age. In addition, patients completed a satisfaction questionnaire at the end of the study related to their participation and treatment regimens.

There were 21 sites in the US that were enlisted, and a total of 16 sites enrolled patients included in the analysis. The study was undertaken in compliance with the protocol, International Council on Harmonisation (ICH) Good Clinical Practice (GCP), and the US Food and Drug Administration (FDA) Code of Federal Regulations. All patients gave written informed consent in accordance with the Declaration of Helsinki. The protocols were approved by the Institutional Review Boards (IRBs) of participating sites.

### Patient Selection and Treatment

The main criteria for inclusion were patients aged ≥ 2 years and ≤ 75 years with a confirmed diagnosis of PIDD as defined by the European Society for Immunodeficiencies (ESID) and Pan-American Group for Immunodeficiency (PAGID) [25] requiring IgRT due to hypogammaglobulinemia or agammaglobulinemia. In this open-label study, cohort assignment was determined per the investigator’s discretion (with input from the subject) and was based on medical history and subject preference. However, subjects who entered the study with dosing frequency already established at every other week were only permitted to enter Cohort 1 or Cohort 2. Patients were also required to be on a consistent or stable dose of any SCIG product for a minimum of 3 months prior to enrollment (patients who entered Cohort 3 had received weekly SCIG infusions for a minimum of 12 weeks).

SCIG 16.5% was administered using syringe drivers at the protocol-specified infusion rate either every week (± 2 days; Cohort 1 and Cohort 2) or every other week (± 2 days; Cohort 3) at double the weekly dose. The dosing frequency was determined by the cohort assignment. Patients received the same mg/kg body weight dose as their previous SCIG product prior to study entry. The dose was calculated by taking the patient’s prior SCIG total dose (mg) and dividing by their weight.

### Statistical Analyses

The collected data were summarized and presented by standard descriptive statistics. In this study, SBIs were defined as bacterial pneumonia, bacteremia/sepsis, osteomyelitis/septic arthritis, visceral abscess, or bacterial meningitis. The rate of SBI per person-year during the SCIG 16.5% treatment period was to be presented as point estimates of the rate. The 2-sided 98% (confidence interval [CI]) – upper limit was to be reported.

For Cohort 3, a confirmatory analysis was performed to evaluate whether the total IgG trough levels were maintained by a bi-weekly dosing regimen. This analysis was performed using a 1-sided, paired t-test at the α = 0.025 level of significance.

Post-hoc analyses were also conducted to determine total serum IgG trough levels < 5 g/dL, hospitalizations due to infection, fever episodes, absences from work or school, and total flow rate and number of infusion sites.

## Results

### Patient Disposition and Demographics

There were 64 patients enrolled in the study who all received treatment (Table 1). A total of 55/64 (85.9%) patients completed the study, with 9/64 (14.1%) who discontinued the study prematurely. The most common reason for discontinuation was decision/withdrawal of consent by the patient (4/64 [6.3%]) (Table 1).

**Table 1:** Summary of Patient Disposition

Patients	Cohort 1: Increased Volume n (%)	Cohort 2: Increased Rate n (%)	Cohort 3: Every Other Week n (%)	Total n (%)
Screened				82
Enrolled	15	15	34	64
Treated	15 (100)	15 (100)	34 (100)	64 (100)
Completed Study	12 (80.0)	13 (86.7)	30 (88.2)	55 (85.9)
Discontinued Study	3 (20.0)	2 (13.3)	4 (11.8)	9 (14.1)
Patient Decision/Withdrawal of Consent	1 (6.7)	2 (13.3)	1 (2.9)	4 (6.3)
Adverse Event	2 (13.3)	0	1 (2.9)	3 (4.7)
Investigator Decision	0	0	1 (2.9)	1 (1.6)
Lost to Follow-up	0	0	1 (2.9)	1 (1.6)

The denominator is the number enrolled; for each cohort, the denominator is the number enrolled in that cohort

In Cohort 1, all 15 patients were adults. In Cohort 2, there was 1 child (aged ≥ 6 and < 12 years) and 1 adolescent (aged ≥ 12 and < 17 years), both of whom completed the study. Of the 34 patients in Cohort 3, there was 1 young child (aged > 2 and < 6 years), 1 child, and 1 adolescent, all of whom completed the study (Table 2). Across all cohorts, the majority of patients were female, White, and not Hispanic or Latino (Table 2). The mean age was approximately 50 years in all cohorts. It should be noted that the pediatric patients in Cohorts 2 and 3 impacted the summaries of height, weight, and body mass index (Table 2). The majority of patients (57/64 [89.1%]) had common variable immunodeficiency (CVID), with a similar percentage across cohorts.

**Table 2:** Summary of Demographic and Baseline Characteristics (Full Analysis Set)

Characteristic	Cohort 1: Increased Volume (N = 15) n (%)	Cohort 2: Increased Rate (N = 15) n (%)	Cohort 3: Every Other Week (N = 34) n (%)	Total (N = 64) n (%)
Age (years)^1^, n	15	15	34	64
Mean (SD)	51.20 (17.27)	47.88 (20.53)	50.81 (18.54)	50.21 (18.49)
Median	49.72	56.52	58.37	55.71
Min, Max	17.2, 74.2	10.5, 67.2	5.7, 71.0	5.7, 74.2
Sex				
Female	10 (66.7)	11 (73.3)	27 (79.4)	48 (75.0)
Male	5 (33.3)	4 (26.7)	7 (20.6)	16 (25.0)
Race				
White	15 (100)	13 (86.7)	34 (100)	62 (96.9)
Multiple	0	2 (13.3)	0	2 (3.1)
Ethnicity				
Not Hispanic or Latino	12 (80.0)	15 (100)	34 (100)	61 (95.3)
Hispanic or Latino	3 (20.0)	0	0	3 (4.7)
ABO Blood Type				
Missing	4 (26.7)	7 (46.7)	18 (52.9)	29 (45.3)
O	9 (60.0)	6 (40.0)	8 (23.5)	23 (35.9)
A	1 (6.7)	2 (13.3)	6 (17.6)	9 (14.1)
B	0	0	2 (5.9)	2 (3.1)
AB	1 (6.7)	0	0	1 (1.6)
Type of PI Disease				
CVID	14 (93.3)	13 (86.7)	30 (88.2)	57 (89.1)
Other^2^	1 (6.7)	1 (6.7)	4 (11.8)	6 (9.4)
XLA	0	1 (6.7)	0	1 (1.6)
Height (cm), n	15	15	34	64
Mean (SD)	169.38 (8.79)	166.65 (14.56)	163.02 (13.28)	165.36 (12.80)
Median	167.50	167.60	165.05	166.20
Min, Max	156.0, 185.4	126.0, 186.0	109.2, 182.9	109.2, 186.0
Weight (kg), n	15	15	34	64
Mean (SD)	100.48 (25.30)	80.03 (23.45)	75.31 (18.84)	82.32 (23.59)
Median	99.60	77.30	79.85	82.45
Min, Max	54.6, 138.9	32.4, 120.0	24.6, 105.6	24.6, 138.9
BMI (kg/m^2^), n	15	15	34	64
Mean (SD)	35.17 (8.99)	28.20 (4.56)	27.98 (5.97)	29.72 (7.11)
Median	32.70	28.20	26.05	28.90
Min, Max	22.5, 49.0	20.6, 34.9	16.5, 38.5	16.5, 49.0

Abbreviations: BMI = body mass index; CVID = common variable immunodeficiency; Max = maximum; Min = minimum; N = number of patients; PI = primary immunodeficiency; SD = standard deviation; XLA = X-linked agammaglobulinemia

^1^Pediatric patients included young children (> 2 and < 6 years), children (≥ 6 and < 12 years), and adolescents (≥ 12 and < 17 years)

^2^ “Other” included 5 cases of hypogammaglobulinemia with antibody deficiency and 1 case of hereditary hypogammaglobulinemia

### Treatment Administration and Extent of Exposure

**Fig. 2:**
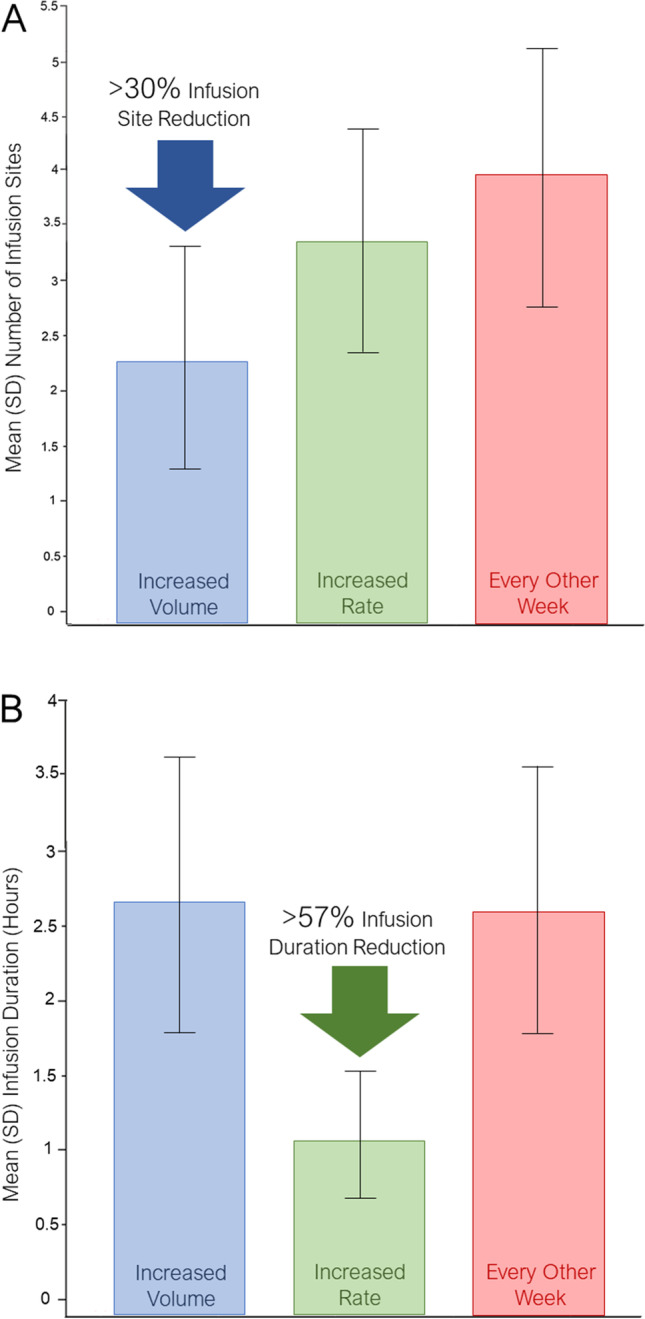
Cohort comparisons of number of infusion sites (**A**) and infusion duration (**B**)

Infusions were administered on-site and at home. There were a total number of 1,338 SCIG 16.5% infusions, which included 386, 396, and 556 administrations in Cohorts 1, 2, and 3, respectively (Table 3). In Cohort 1 (15 adult patients treated with increased infusion volume up to a maximum of 100 mL/infusion site), the mean (standard deviation [SD]) maximum volume per site was 69.43 (23.47) mL/site ranging from 36.0 to 108 mL/site) (Table 3). The mean (SD) volume administered per infusion was 83.38 (21.82) mL over a mean of 2.3 infusion sites. Due to the increased volume per site, the number of infusion sites was reduced in Cohort 1 as compared to the other cohorts resulting in fewer overall needle sticks for patients (Fig. 2A). One-third of patients (5/15; 33.3%) attained ≥ 90% of the allowed maximum volume of 100 mL/site, a further third attained between 50% and < 90% of the allowed maximum, and one third attained < 50% of the allowed maximum.

**Table 3:** Summary of Treatment Administration and Exposure (Safety Analysis Set)

	Cohort 1: Increased Volume (N = 15)	Cohort 2: Increased Rate (N = 15)	Cohort 3: Every Other Week (N = 34)	Total (N = 64)
Actual SCIG 16.5% Dose Administered per Body Weight (g/kg)^a^				
Mean (SD)	0.146 (0.05)	0.157 (0.07)	0.257 (0.10)	0.195 (0.09)
Median	0.132	0.150	0.256	0.175
Min, Max	0.06, 0.23	0.08, 0.32	0.02, 0.47	0.02, 0.47
Total Volume Administered per Infusion (mL)				
Mean (SD)	83.38 (21.82)	70.82 (27.83)	117.61 (55.19)	93.88 (46.67)
Median	90.00	60.00	108.00	90.00
Min, Max	42.0, 133.0	38.0, 144.0	7.0, 252.0	7.0, 252.00
Duration of All Infusions – Active Time Only (hours)				
Mean (SD)	2.65 (1.16)	1.10 (0.77)	2.61 (1.27)	2.17 (1.31)
Median	2.39	0.87	2.33	2.00
Min, Max	0.9, 7.6	0.3, 5.1	0.3, 7.7	0.3, 7.7
Infusion Sites Used per Infusion				
Mean (SD)	2.3 (1.05)	3.3 (1.07)	3.9 (1.24)	3.3 (1.33)
Median	2.0	3.0	4.0	3.0
Min, Max	1, 6	2, 5	1, 6	1, 6
Total Number of Infusions per Patient, n				
Mean (SD)	25.7 (6.81)	26.4 (7.08)	16.4 (2.63)	20.9 (7.00)
Median	29.0	29.0	17.0	17.0
Min, Max	6, 30	8, 30	7, 21	6, 30
Maximum Realized Volume per Site: All Infusions (mL/site)^c^, n				
Mean (SD)	69.43 (23.47)	25.25 (10.93)	35.13 (11.42)	40.85 (22.08)
Median	72.00	24.00	34.80	36.00
Min, Max	36.0, 108.0	10.5, 48.0	16.3, 66.0	10.5, 100.0
Maximum Realized Flow Rate per Site: All Infusions (mL/h/site)^c^, n				
Mean (SD)	32.92 (17.93)	42.06 (13.02)	19.25 (7.09)	27.80 (15.15)
Median	26.47	42.86	19.30	23.79
Min, Max	14.5, 81.5	17.1, 67.5	10.1, 37.3	10.1, 81.5

Abbreviations: Max = maximum; Min = minimum; N = number of subjects; SCIG = subcutaneous immunoglobulin; SD = standard deviation

^a^Per body weight units were calculated by dividing each subject’s dose by baseline weight (kg)

^b^n was the number of infusions rather than subjects

^c^Maximum realized volume and flow rate were calculated for each patient as maximum of realized volume and flow rate over all infusions

In Cohort 2 (13 adult patients and 1 child and 1 adolescent treated with increased infusion flow rates up to a maximum of 100 mL/h/site or 240 mL/h for all sites combined), the mean volume administered per infusion was 70.82 mL (range: 38.0 to 144.0 mL) over a mean of 3.3 infusion sites. In the adult patients, the mean (SD) volume per infusion was 75.42 (27.57 mL (same range as above). The mean (SD) maximum realized flow rate per site was 42.06 (13.02) mL/h/site ranging from 17.1 to 67.5 mL/h/site. Due to the increased flow rate, the mean infusion duration was decreased by > 57% as compared to the other cohorts (Fig. 2B). Results demonstrated that 60% of patients attained between 50 and 75% of the allowed maximum flow rate of 240 mL/h, 13.3% attained between 75 and 90% of the allowed maximum, and 26.7% attained < 50% of the allowed maximum.

In Cohort 3 (31 adult patients, 1 young child, 1 child, and 1 adolescent with a switch to dosing every other week with no protocol-defined increases to flow rate or volume), the mean (SD) volume administered per infusion was 117.61 (57.84) mL (range: 7.0 to 252.0 mL) over a mean of 3.9 infusion sites, and the maximum realized mean (SD) flow rate per site was 19.25 (7.01) mL/h/site.

### Efficacy

There were no IgG trough levels that were < 5 g/L, which is recognized as the minimum serum IgG concentration needed to provide protection from infection. Studies have shown that PIDD patients with higher IgG trough levels are less likely to experience infections and may have better long-term outcomes than those with lower levels [26–28]. Additionally, higher IgG levels may be necessary for patients with more severe forms of primary immune deficiency, as they may be at greater risk for infections and other complications [26–28].

**Fig. 3:**
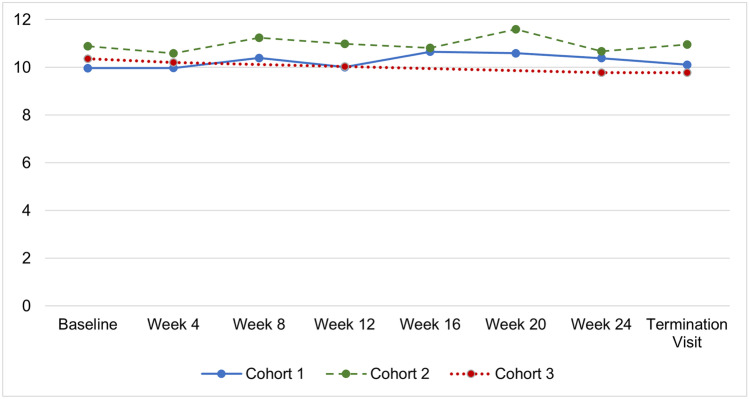
Mean immunoglobulin G trough levels by cohort from baseline to the termination visit

In Cohorts 1 and 2, mean values were generally slightly increased from baseline between Week 8 through Week 24, and decreased from baseline for Cohort 3 at Week 12 and Week 24. In addition, in Cohort 3, a decrease in mean (SD) total IgG trough levels was seen with every other week dosing (9.927 [2.01] g/L) compared to weekly dosing (10.364 [1.96] g/L) (p = 0.0017;1-sided 97.5% lower confidence limit [LCL] = 0.799, Infinity), but it was not considered to be clinically meaningful (Table 4 and Fig. 3).

**Table 4:** Summary of Total IgG Trough Levels Overall and in Adults – Cohort 3 (Full Analysis Set and Per-Protocol Set)

Immunoglobulin G (g/L)	Dosing Regimen (Full Analysis Set)		Dosing Regimen (Per-Protocol Set)	
	Weekly	Every Other Week	Weekly	Every Other Week
Overall, n	34	34	32	32
Mean (SD)	10.364 (1.96)	9.927 (2.01)	10.449 (1.99)	9.959 (2.06)
Median	10.075	9.945	10.275	9.945
Min, Max	7.24, 15.18	6.23, 15.49	7.24, 15.18	6.23, 15.49
1-sided 97.5% LCL		-0.799, Infinity		-0.866, Infinity
*p*-value		0.0017		0.0048
Adults ≥ 17 years, n	31	31	29	29
Mean (SD)	10.331 (1.93)	9.955 (2.05)	10.422 (1.96)	9.992 (2.10)
Median	9.980	10.030	10.170	10.030
Min, Max	7.24, 15.18	6.23, 15.49	7.24, 15.18	6.23, 15.49
1-sided 97.5% LCL		-0.761, Infinity		-0.834, Infinity
*p*-value		0.0012		0.0037

Abbreviations: CI = confidence interval; IgG = immunoglobulin G; LCL = lower confidence limit; Max = maximum; Min = minimum; NA = not applicable; SD = standard deviation

There were no SBIs reported during the study. There were a total of 63 infections reported in 37/64 (57.8%) patients. Almost all patients with infections were adults. Infections were reported in 2 children, both in Cohort 3. The mean (SD) rate of infections per person-year overall was 2.14 (2.73; 98% CI: 1.34, 3.51) and was slightly higher in Cohorts 1 and 2 (3.16 and 2.22 infections per person-year, respectively) than Cohort 3 (1.66 infections per person year) (Table 5).

**Table 5:** Summary of Infection Rates per Year in the Treatment Period (Full Analysis Set)

Any Infection	Cohort 1: Increased Volume (N = 15)	Cohort 2: Increased Rate (N = 15)	Cohort 3: Every Other Week (N = 34)	Total (N = 64)
Treatment Period				
Patients with any infection during Treatment Period, n (%)	10 (66.7)	8 (53.3)	19 (55.9)	37 (57.8)
Number of infections during Treatment Period, n	22	14	27	63
Duration of Treatment Period (Annualized), n	15	15	34	64
Mean (SD)	0.42 (0.13)	0.44 (0.13)	0.47 (0.11)	0.45 (0.12)
Median	0.48	0.48	0.50	0.49
Min, Max	0.0, 0.5	0.1, 0.5	0.1, 0.6	0.0, 0.6
Rate of infections per person-year, n	15	15	34	64
Mean (SD)	3.16 (4.08)	2.22 (2.56)	1.66 (1.92)	2.14 (2.73)
Median	2.09	2.02	1.94	1.99
Min, Max	0.0, 16.0	0.0, 6.4	0.0, 6.3	0.0, 16.0
98% CI	1.28, 9.35	0.86, 5.33	0.90, 3.10	1.34, 3.51

Abbreviations: CI = confidence interval; Max = maximum; Min = minimum; SD = standard deviation

A total of 39/64 (60.9%) patients were treated with antibiotics during the study. Out of the total number of patients treated with antibiotics, 36/64 (56.3%) received systemic antibiotics and 6/64 (9.4%) received topical antibiotics. Of the patients who received topical antibiotics, 3/64 (4.7%) received both systemic and topical antibiotics while the remaining 3 patients only received topical antibiotics. Topical antibiotics were utilized for the treatment of infusion site abscess, left shin abrasion, left foot laceration, left foot infection, methicillin-resistant Staphylococcus aureus of the left buttock, and folliculitis.

The median number of treatment episodes annualized was 1.73 episodes for both antibiotic and systemic antibiotic use. The annualized median number of treatment days for antibiotics and systemic antibiotics was 17.07 days and 10.37 days, respectively, and was higher in Cohort 1 than Cohorts 2 and 3 for both. There was 1 (1.6%) patient who was hospitalized due to a coronavirus disease 2019 (COVID-19) infection in the study; this patient was in the adult age group, in Cohort 3, and was hospitalized for a total of 5.0 days.

There were 8 episodes of fever reported among 4 (6.3%) patients (including 1 child), for an annualized rate of 0.23 episodes of fever per year. The total number of episodes of fever per year was higher in Cohort 2 (0.52 episodes) than Cohorts 1 and 3 (0.13 and 0.16 episodes, respectively). Overall, 11 absences from work or school due to infections were reported among 5/64 (7.8%) patients (all adults) for a total of 24 days of absence. The median rate of absence from work or school per person-year was 0.18, assuming 200 working/school days per year.

The analysis of SF-10 scores included questionnaire responses completed by the 3 pediatric patients (≤ 13 years of age). There was 1 child in Cohort 2 with increases in the physical and psychosocial summary scores (indicating improved QoL) and 1 young child and 1 child in Cohort 3 with no marked changes in QoL. The analysis of SF-36 scores included responses from 61 patients (≥ 14 years of age). The changes in rate, volume, and frequency were well tolerated and did not result in any deterioration of QoL.

### Safety

Overall, there were 420 TEAEs experienced by 55/64 (85.9%) patients, which included 95 events in 13/15 (86.7%), 171 events in 12/15 (80.0%), and 154 events in 30/34 (88.2%) patients in Cohorts 1, 2, and 3, respectively. The most commonly reported TEAEs overall were infusion site erythema (31.3%), infusion site pruritus and sinusitis (both 23.4%), with a similar profile for each cohort, except headache was also more commonly reported in Cohort 3 (20.6% for Cohort 3 and 15.6% overall). There were 77 infection-only TEAEs in 43/64 (67.2%) patients and 161 ISR-only TEAEs in 31/64 (48.4%) patients. Cohort 1 had the highest proportion of patients with infection-only TEAEs (80.0%), while the proportion of ISR-only TEAEs was higher in Cohorts 1 and 2 (53.3% for both) (Table 6). TEAEs were experienced by 51 adult patients (396 events), 2 adolescents (2 events), 1 child (18 events), and 1 young child (4 events).

**Table 6:** Summary of Treatment-Related, Temporally-Associated Treatment-Emergent Adverse Events, Including Infusion Site Reactions, in ≥ 5% of Patients in the Treatment Period (Safety Analysis Set)

System Organ Class Preferred Term	Cohort 1: Increased Volume (N = 15)		Cohort 2: Increased Rate (N = 15)		Cohort 3: Every Other Week (N = 34)		Total (N = 64)	
	n (%)	# of Events	n (%)	# of Events	n (%)	# of Events	n (%)	# of Events
Any systemic related temporally-associated TEAE	9 (60.00)	24	8 (53.3)	85	13 (38.2)	51	30 (46.9)	160
General disorders and administration site conditions	9 (60.0)	24	8 (53.3)	72	10 (29.4)	30	27 (42.2)	126
Infusion site erythema	4 (26.7)	7	8 (53.3)	43	4 (11.8)	5	16 (25.0)	55
Infusion site pruritus	4 (26.7)	6	4 (26.7)	14	4 (11.8)	8	12 (18.8)	28
Infusion site pain	1 (6.7)	1	3 (20.0)	5	2 (5.9)	4	6 (9.4)	10
Nervous system disorders	0	0	4 (26.7)	7	4 (11.8)	9	8 (12.5)	16
Headache	0	0	3 (20.0)	4	4 (11.8)	9	7 (10.9)	13

Abbreviation: TEAE = treatment-emergent adverse event

Within each category, patients were counted only once if they had more than one event

Adverse evets were coded using MedDRA version 24.1

The majority of patients reported mild (23.4%) or moderate (56.3%) TEAEs. There were 4/64 (6.3%) patients (all adults) with severe TEAEs (all Grade 3; no Grade 4 or 5), which included 2/15 (13.3%) and 2/34 (5.9%) patients in Cohorts 1 and 3, respectively. Severe TEAEs were infusion site abscess (unlikely related to study drug) in Cohort 1, and abdominal distension (probably related to study drug) and COVID-19 (unrelated to study drug) in Cohort 3. No TEAEs led to death. There were 3/64 (4.7%) patients (1 in each cohort; all adults) who experienced treatment-emergent SAEs of COVID-19 (severe), dehydration (severe), and rheumatoid arthritis (moderate), which were all considered unrelated to study drug.

During the treatment period, systemic treatment-related, temporally associated TEAEs were experienced by 30/64 (46.9%) of patients (Table 6). General disorders, including infusion site reactions, were experienced by 27/64 (42.2%) of patients, while 7/64 (10.9%) experienced headache (Table 6).

### Treatment Compliance and Patient Experience

Treatment was performed either on-site or at home. Overall, 24/64 (37.5%) patients had 176 (13.2%) infusions that deviated from the scheduled administration interval by more than 2 days and 14/64 (21.9%) patients had 46 (3.4%) infusions administered at an incorrect dose. Cohort 2 had the lowest number of subjects with deviations in their scheduled treatment. Across all cohorts, there were 23 on-site infusions in 17 patients that were impacted by interruptions, which were primarily due to technical difficulties with infusion lines and pump settings, incorrect dosing, change of syringes, infusion related reaction, and needle and insertion site leakages. Only 1 patient had their on-site infusion stopped as it was discovered that they had already infused at home on a prior date. Despite the increased infusion parameters, only 3 patients (4.7%) withdrew from the study as a result of adverse events.

**Fig. 4:**
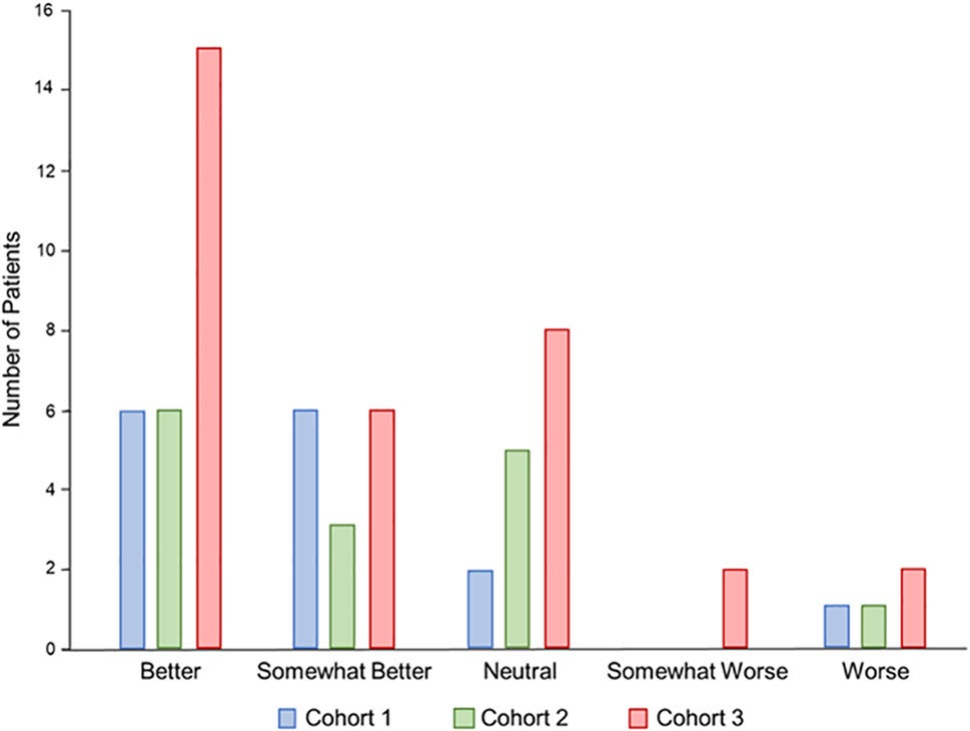
Patient satisfaction scores by cohort

A satisfaction questionnaire was completed at the Termination Visit to record the patient’s opinion related to their participation in the study. The majority of patients (67.3%) reported that they found the new infusion regimen to be better or somewhat better than their previous regimen and that switching from their previous SCIG product to SCIG 16.5% was very easy. Overall and across cohorts, the majority of patients reported to have either the same or fewer illnesses and/or AEs compared to their previous SCIG product (Fig. 4).

## Discussion

For patients with PIDD, IgRT is typically a life-long treatment. Over time, SCIG infusions become routine, and compliance, time, and convenience become increasingly important factors to improve patient experience. Therefore, it is very important to explore improvements in the practical usage of these therapies in meeting not only clinical requirements but also the patients' lifestyle, daily routine, and impact on their overall quality of life. This protocol evaluated study patients receiving weekly doses of SCIG 16.5% at increased infusion volumes to reduce infusion sites, increased flow rates to reduce the duration of infusions, or receiving every other week infusions at double the weekly dose over a period of up to approximately 6 months. Previous clinical studies [7, 8] have provided guidance on dosing of SCIG 16.5% when switching PIDD patients from IVIG to SCIG. The current study was designed to evaluate enhanced infusion regimens which saved time, reduced infusion sites and associated needle sticks, and reduced frequency which may lead to increased patient compliance and treatment satisfaction by allowing for greater administration flexibility.

It is noteworthy that the rate and intensity of TEAEs did not increase with enhanced infusion parameters. Overall, the evaluation of TEAEs and other safety parameters indicate that SCIG 16.5% at enhanced infusion regimens (i.e., increased infusion volume in Cohort 1, increased infusion rate in Cohort 2, or every other week dosing in Cohort 3) was well tolerated. The majority of TEAEs were considered to be mild or moderate in severity, with only 4 severe TEAEs reported overall. There were no deaths and only a small number of treatment-emergent SAEs (3 in total; 1 in each cohort), and all SAEs were assessed as unrelated to study treatment. Additionally, only 3 patients discontinued the study due to TEAEs (2 in Cohort 1 and 1 in Cohort 3; see Table 1).

The most common TEAEs were ISRs, specifically infusion site erythema and infusion site pruritus. The majority of ISRs reported during the study were mild or moderate in severity. The proportion of patients with ISRs were similar between weekly infusions with increasing infusion volumes (Cohort 1; 8/15 [53.3%] patients) and increasing infusion flow rates (Cohort 2; 8/15 [53.3%] patients) and slightly lower with every other week infusions (Cohort 3; 15/34 [44.1%] patients). Interestingly, the proportion of patients experiencing ISRs in the pivotal and extension studies was 59/81 (73%); thus, the number and rate of ISRs decreased in this study despite increasing infusion parameters [22].

There were no SBIs during the study, indicating that SCIG 16.5% administered via the 3 enhanced infusion regimens was effective in preventing the occurrence of SBIs in patients with PIDD. The mean rate of non-SBI infections per person-year was 2.14 (98% CI: 1.34, 3.51), which is again lower than the total rate of non-SBI infections reported in the pivotal and extension studies (2.2, 95% CI: 2.4, 4.5).

Patients in Cohort 3 were on a weekly infusion regimen for a minimum of 12 weeks prior to entering the study, and then received their infusions every other week for 24 weeks. Differences in IgG levels at baseline compared to the end of treatment were not considered clinically meaningful. Serum IgG trough levels remained relatively constant throughout the study.

The concept of autonomy has gained widespread recognition as a crucial aspect of contemporary healthcare practice [29, 30]. Freedom of choice is closely linked to the notion of personal sovereignty. In the context of medical treatment, it is widely held that empowering patients with choices promotes their independence, enhances their understanding and personal responsibility for their health condition, and has been linked to their satisfaction [29, 30]. In this study, the satisfaction scores reflect that the majority of patients found the new infusion regimen to be better or somewhat better than their previous regimen and that switching from their previous SCIG product to SCIG 16.5% was very easy. Giving patients the flexibility to choose the best infusion regimen based on their current lifestyle, circumstances, and preferences, with equivalent safety and efficacy, enhances their sense of control and autonomy over their health and treatment.

## Conclusions

In summary, the results of the trial demonstrate that enhanced SCIG 16.5% (Cutaquig®) infusions are efficacious and safe at higher infusion parameters and every other week treatments. Increasing rates of infusion volume resulted in a > 30% reduction of injection sites and associated needle sticks, while increasing infusion rates decreased infusion duration by > 57%. Dosing every other week demonstrated equivalency to IgG trough levels with weekly dosing. In addition, the majority of patients found the new infusion regimens to be better or somewhat better than their previous regimens. Thus, the results from this study provide patients and physicians with more options to facilitate SCIG 16.5% infusions.

## Data Availability

Data for this study will be made available per request.
